# Epilepsy Treatment Outcome and Its Predictors among Ambulatory Patients with Epilepsy at Mizan-Tepi University Teaching Hospital, Southwest Ethiopia

**DOI:** 10.1155/2020/8109858

**Published:** 2020-04-08

**Authors:** Ameha Zewudie, Yitagesu Mamo, Desalegn Feyissa, Mohammed Yimam, Gosaye Mekonen, Ahmed Abdela

**Affiliations:** ^1^Department of Pharmacy, College of Medicine and Health Science, Mizan-Tepi University, Mizan-Aman, Ethiopia; ^2^Department of Pharmacy, College of Medicine and Health Science, Ambo University, Ambo, Ethiopia; ^3^Department of Pharmacy, College of Health Science, Bule-Hora University, Bule-Hora, Ethiopia

## Abstract

**Background:**

Epilepsy is among the most common neurological disorders which is highly treatable with currently available antiepileptic drugs at a reasonable price. In Ethiopia, despite a number of studies revealed high prevalence of epilepsy, little is known on predictors of poorly controlled seizures. Thus, the aim of this study was to assess epilepsy treatment outcome and its predictors among patients with epilepsy on follow-up at the ambulatory care unit of Mizan-Tepi University Teaching Hospital, Southwest Ethiopia.

**Methods:**

A hospital-based cross-sectional study involving patient interview and chart review was conducted from March 10 to April 10, 2018. Drug use patterns and sociodemographic data of the study participants were accustomed to descriptive statistics. Backward logistic regression analysis was done to identify predictors of poor seizure control. Statistical significance was considered at *p* value <0.05.

**Results:**

From a total of 143 studied patients with epilepsy, 60.8% had uncontrolled seizures. Monotherapy (79%) was commonly used for the treatment of seizures, of which phenobarbital was the most commonly utilized single anticonvulsant drug (62.9%). The majority (72.7%) of the patients had developed one or more antiepileptic-related adverse effects. Medium medication adherence (adjusted odds ratio (AOR) = 5.4; 95% CI = 1.52–19.23; *p*=0.009), poor medication adherence (AOR = 8.16; 95% CI = 3.04–21.90; *p*=0.001), head injury before seizure occurrence (AOR = 4.9; 95% CI = 1.25–19.27; *p*=0.02), and seizure attacks ≥4 episodes/week before AEDs initiation (AOR = 8.52; % CI = 2.41–13.45; *p*=0.001) were the predictors of uncontrolled seizure.

**Conclusions:**

Based on our findings, more than half of the patients with epilepsy had poorly controlled seizures. Nonadherence to antiepileptic drugs, high frequency of seizure attack before AEDs initiation, and history of a head injury before the occurrence of seizure were predictors of uncontrolled seizure. Patient medication adherence should be increased by the free access of antiepileptic drugs and attention should be given for the patients with a history of head injury and high frequency of seizure attacks before AEDs initiation.

## 1. Background

Epilepsy is a noncommunicable disease of the brain that affects all communities with unequal distribution. About 10% of the entire world population living a normal life span can expect to have at least one epileptic seizure. There are about 65 million patients with epilepsy worldwide, of whom 80% are living in developing countries [1]. This could be underestimated because partial seizures are often underdiagnosed in the less developed world. If these patients have been treated appropriately with AEDs, 70% of them could be seizure-free [1, 2].

About 90% of people with epilepsy in Africa were untreated despite the fact that highly cost-effective treatments were available [3]. Although currently available therapies with AEDs can effectively treat the majority of newly diagnosed patients, nonadherence to therapy and inappropriate use of AEDs may significantly affect seizure control [4]. Adequate patient adherence to AEDs, avoiding any seizure triggering factors, and proper patient education about their diseases and management have been implicated for better seizure control [5].

The proportion of deaths that are epilepsy-related may be much higher in Africa than in other regions of the world. In a series of 164 patients with epilepsy recruited from a rural clinic in Tanzania, 67.1% of them died and the mortality rate was twofold that of the Tanzanian rural population. In greater than 50% of the patients, the causes of death were related to epilepsy [6].

Epilepsy accounts for 0.6% of all diseases globally, as a result of years of life lost due to early mortality and time lived with a disability. It has a high economic burden in terms of health care needs, early death, and unproductivity due to absenteeism. A study conducted in India revealed that the cost per patient for epilepsy treatment was as high as 88.2% of the Indian's per capita gross national product (GNP). This epilepsy-related cost includes travel cost, lost work time, and medical costs which exceeded $2.6 billion/year [7].

To solve the problem, the World Health Assembly (WHA) adopted a resolution called the burden of epilepsy globally and at countries level to address its health, public knowledge, and social implications. This resolution advocates governments to formulate, strengthen, and implement their policies to facilitate access to care and protect the right of patients with epilepsy. It focuses on the relevance of training general practitioners and other health care providers in order to reduce epilepsy treatment gaps [7]. Regardless of these interventions, epilepsy is poorly controlled all over the world particularly in developing countries.

To our knowledge, there is no study in Southwest Ethiopia on predictors of poorly controlled seizure. Moreover, studies conducted on determinants of poorly controlled seizures in North Ethiopia had major limitations. In those studies, a controlled seizure was defined as if the patients were seizure-free for 6 months or 1 year. This definition does not fulfill the World Health Organization's (WHO) definition of controlled seizure. In our study, patients with epilepsy were assessed if they were on AEDs for at least 2 years. Seizures are controlled if the patients were seizure-free for at least 2 years. Hence, this study aimed to assess epilepsy treatment outcome and its predictors among patients with epilepsy on follow-up at the ambulatory care unit of Mizan-Tepi University Teaching Hospital (MTUTH), Southwest Ethiopia.

## 2. Methods

### 2.1. Study Design and Setting

A hospital-based cross-sectional study was conducted in MTUTH which is found in Mizan-Aman Town and located at 561 km away from the capital city of Ethiopia, Addis Ababa. The study was conducted from March 10 to April 10, 2018.

### 2.2. Study Participants

The source of the population was all epileptic patients attending the ambulatory unit of MTUTH, while the study population consists of all epileptic patients who were attending the ambulatory unit of MTUTH during the study period and fulfilled inclusion criteria. Patients (age ≥15 years) with a diagnosis of epilepsy who have been taking AEDs and on regular follow-up for at least two years were included in our study. The participants were interviewed, and their medical records were reviewed during their appointment for medication refilling. They were excluded from the study if their medical records were with incomplete data, if they were mentally unstable (such as aggressive patients, critically ill patients, patients with acute psychosis, panic attack, or status epilepticus), or if they were patients who were acute sick looking (determined by clinical presentation) and refused to give consent, and if they were admitted to the hospital during data collection. Among 182 epileptic patients who have been on follow-up at the ambulatory unit of MTUTH, 143 epileptic patients met inclusion criteria. All patients who fulfilled the inclusion criteria were selected as the study participants.

### 2.3. Data Collection Procedure, Variables, and Outcome of the Study

All epileptic patients who have been on follow-up at the hospital during the data collection period and fulfill inclusion criteria were included in the study. Data abstraction format was used to retrieve patients' clinical information and medication experience such as the status of treatment outcome, types of epilepsy, seizure frequency, duration of follow-up, comorbidity, types of AEDs, and AEDs-related adverse effects from patients' medical record. A semistructured questionnaire was used to collect patients' sociodemographic data, history of head injury, triggering factors, and medication adherence. Medication adherence was assessed using a self-reported questionnaire which was developed based on the review of literatures [8–11]. Patients were asked whether or not they had missed or stopped a dose of their AED for any reason in the last one month. We assessed the adherence level in the last one-month period because of published literatures supporting the one-month recall period [8, 12, 13]. Accordingly, epileptic patients who took their AEDs without missing any dose in the last month were considered as high adherence. Epileptic patients who missed 1 dose of their AEDs in the last month were said to be medium adherence, whereas patients who missed 2 or more doses were considered as low adherence to AEDs.

Since we have no data on definite diagnosis for the type of epilepsy except generalized tonic-clonic seizure, and genetic variability between the participants, these variables can be considered as potential confounders. Epilepsy treatment outcome was assessed in terms of seizure control and seizure frequency. The seizure status was considered to be well controlled if the patients had not experienced any seizure episode in the last two years, and poorly controlled if they experienced one or more seizure episodes in the last two years of follow-up period.

### 2.4. Statistical Analysis

The data were entered into Epidata manager version 4.0.2.101 and analyzed by Statistical Package for Social Science (SPSS) version 21. Bivariate logistic regression was done to see the association between independent variables and seizure control. Variables with *p* value ≤0.25 on bivariate logistic regression were entered into multivariate logistic regression. Multivariable logistic regression analyses using backward selection were done to identify predictors of poor seizure control at *p* value of <0.05 significant.

## 3. Result

### 3.1. Sociodemographic Characteristics of the Study Subjects

There were 182 ambulatory epileptic patients at the epilepsy unit of MTUTH. Of them, 9 patients refused an interview, 17 patients were under 15 years, and 13 patients' medical charts were incomplete. One hundred and forty-three participants were interviewed, their medical records were reviewed, and analysis was done. Among the participants, 48.3% were female. Most patients (86.8%) were below 45 years of age and more than half (54.6%) of the patients' ages fell between 15 and 30 years. More than half (55.2%) of the participants attended primary school and about 37.1% of epileptic patients were students (Table 1).

### 3.2. Clinical Information and Determinants of Prognostic Factors

Among the studied participants, 39.1% were seizure-free while 60.8% of them had one or more seizure episodes during the last 2 years. Generalized tonic-clonic seizure (GTCS) was the most commonly diagnosed type of epilepsy (77.6%). About 80.5% of studied participants had 2–5 years of follow-up period and 28.0% had one or more comorbidities. A psychiatric disorder was (13.3%) the most commonly identified comorbidity. About 73.4% of the studied patients with epilepsy had one or more seizure precipitating factors and less than half (44.0%) of them suffered from emotional stress as a seizure triggering factor. Among the studied participants, 47.5% had a history of brain injury; of them, 37.9% had a brain injury before seizure occurrence (Table 2).

### 3.3. Medication Experience of Epileptic Patients

The initial AEDs prescribed for patients with epilepsy were phenobarbitone (81.8%), phenytoin (15.42%), and carbamazepine (2.88%). Among AEDs, the most commonly used drug as add-on therapy for the patients whose seizure was not controlled was phenytoin (14.7%). Phenobarbital was prescribed for 81.8% of the participants and 21% of the patients had one or more antiepileptic drugs. The majority (72.7%) of the patients with epilepsy had developed one or more antiepileptic-related adverse effects. More than half (54.5%) of the studied participants had developed sedation. The other AEDs-related adverse effects were confusion (7.0%), weakness (6.3%), gingival hyperplasia (6.3%), rash (4.9%), blurred vision (4.2%), and gastrointestinal irritation (GI) (2.8%) (Table 3).

### 3.4. Adherence Status and Reasons for Nonadherence

Among 143 studied participants, 58.7%, 16.8%, and 24.5% had low, medium, and high adherence to AEDs, respectively. The most common reasons for nonadherence were forgetfulness (43.5%) and unaffordability (40.7%). Lack of education about AEDs (31.5%), long distance from treatment setting (27.8%), unscheduled hospital follow-up due to workload (21.3%), high cost of medication (19.4%), and other less common factors were reported by the patients (Table 4).

### 3.5. Predictors of Poor Treatment Outcome

All variables with *p* value <0.25 in bivariate logistic regression were entered into multivariate logistic regression to control confounding variables. After adjusting for the other variables, medium adherence to AEDs (AOR = 5.40; 95% CI = 1.52–19.23; *p*=0.009), low adherence to AEDs (AOR = 8.16; 95% CI = 3.04–21.90; *p*=0.001), history of head injury before seizure occurrence (AOR = 4.90; 95% CI = 1.25–19.27; *p*=0.02), and seizure attack >4 episodes/week (AOR = 1.98; 95% CI = 1.053–5.978; *p*=0.012) before AEDs initiation were independent predictors of poorly controlled seizure (Table 5).

## 4. Discussion

The current study aimed to assess epilepsy treatment outcome and its predictors among patients with epilepsy who had been on follow-up at the ambulatory clinic of MTUTH, Southwest Ethiopia.

This study revealed that the most common type of seizure was GTCS. The reason for this might be multifactorial. Knowing the two main semiological components of seizures (physiologic and autonomic) is the basic step in exactly diagnosing the type of seizures. For instance, GTCS may present with violent body movements and often prominent autonomic changes. As a result, the health care seeking in those populations is higher than other types of seizures [14]. This argument is also supported by a study conducted in Ethiopia which showed that 95.1% of patients with epilepsy seek treatment if there is a sudden loss of consciousness [15]. In contrast to this, the partial or focal seizure had been often underdiagnosed in a developing country, because of different reasons [1, 2]. For instance, unlike that of GTCS, in focal seizure, disturbance of cognition is inapparent and loss of consciousness is not usual [14]. Even though traumatic brain injury is a known cause of focal/partial seizure, most of the symptoms associated with simple partial seizure are internal and only noticed by the person having the seizure, another hindering factor for clinical diagnosis and for seeking health care [16]. Thus, such type of seizure may be considered as an unclassified type of seizure. Therefore, these might be the reasons for why GTCS was the most common type of seizure compared to the other seizure types in our study.

In this study, the most commonly prescribed AED as monotherapy was phenobarbitone (81.8%) followed by phenytoin (15.4%). This is in line with the studies conducted in Jimma and Gonder, Ethiopia [17, 18]. In contrast to this, a study done in the United Kingdom reported that the most commonly used AED was carbamazepine (37.4%) followed by sodium valproate (35.7%) [19]. This might be due to the difference in sociodemographic characteristics of the patients and a large sample size used in the previous study.

The majority of the patients were on monotherapy and only one-fourth of them were on polytherapy. The most commonly used AEDs as polytherapy were the combination of phenobarbitone with phenytoin (11.9%) followed by the combination of the phenobarbitone and carbamazepine (6.3%). In contrast to this, studies done in different parts of India revealed that either phenytoin or carbamazepine was the most prescribed AED as monotherapy [20, 21].

Although optimum AED therapy eliminates seizure by 70%, approximately one-third of patients continue to experience seizures despite appropriate treatment [22, 23]. But, our finding was contrary to these reports; only 39% of patients with epilepsy were seizure-free during the last two years. This figure is lower as compared with the study done in different parts of the country [18, 21]. The reason could be the difference in the duration of the outcome measurement report, which was short in the previous study. For instance, the study done in Gonder and Mekele measured the treatment outcome after the patients took AEDs for 3 months and 1 year, respectively [18, 24]. But, in the current study, the status of seizure was measured for the epileptic patients on AEDs for at least 24 months. The second reason might be level of adherence. Studies showed that 70% of controlled seizure was reported for patients with optimum or good adherence level to AEDs [22, 23]. In our study, majority of the patients were nonadherent to AEDs.

In this study, the most commonly diagnosed comorbidity among studied patients with epilepsy was found to be a psychiatric condition (13.3%). This is similar to the study done in the USA (33.7%) and Mekelle, Ethiopia (20.37%) where the psychiatric condition was the most common comorbidity [24, 25].

In the present study, the majority of the patients had developed AEDs-related adverse effects (72.7%). This is higher than the studies done in Mekelle and Ambo, Ethiopia, where 43.3% and 41.7% of patients experienced AEDs-related adverse effects, respectively [24, 26]. The discrepancy might be due to the fact that, in the current study, some of the patients were diagnosed with an unclassified type of seizures which might result in incorrect dose, frequency, drug selection, and drug interaction which in turn cause adverse drug effects. The other probable reason might be due to the fact that, in our study, the studied participants were exposed to AEDs for the duration of ≥2 years whereas ≤1 year in the previous studies.

Our findings indicated that level of adherence, history of a head injury before seizure occurrence, and frequency of seizure attacks before AEDs initiation were found to be determinant factors of epilepsy treatment outcome. The result of this study showed that low medication adherence was an independent predictor of uncontrolled seizures. This is in line with the studies done in Gonder and Mekelle, Ethiopia [18, 24]. In addition, this study showed that high adherence to AEDs decreases the risk of seizure attacks [27]. In this study, the most common reason for nonadherence was forgetfulness (43.5%). In a similar way, the study done in China indicated that 69.6% of the participants had forgotten to take their AEDs [23]. Moreover, a study conducted in Ethiopia previously revealed that forgetfulness was the main reason for nonadherence [28].

Even though the majority of our study participants have been taking phenobarbital, AED with long duration of action, it was found that missing one dose per month (medium medication adherence) was an independent predictor of poor seizure control. The possible reason that justifies the association might be self-reporting-related bias. Study indicated that underreporting of adherence level (self-reporting-related bias) and trying to reduce number of missed medications to get acceptance from health care providers is the main problem of measuring adherence [29].

Studies revealed that uncontrolled seizure was more likely among individuals who had a history of head injury than those who had no history of a head injury before seizure occurrence [18, 30]. Similarly, head injury before diagnoses of epilepsy was found to be the determinant of uncontrolled seizures in this study. Since this study is a cross-sectional study, it cannot address the causal effect. Study indicates that seizures are a common complication of head injuries [6]. In contrast to the current study, a study conducted in Ambo, Ethiopia, showed that there is no significant association between head injury and uncontrolled seizure [26].

In our study, a high frequency of seizure episodes (greater than or equal to four seizure attacks per week before AED initiation) was the other independent predictor of uncontrolled seizures. To our knowledge, there is only one study researched with the variable “number of seizure attacks per week before AEDs initiation” as an independent variable. In this study, there is an association between the number of seizure attacks per week before AEDs initiation and uncontrolled seizure on bivariate analysis. But the author did not run multivariate analysis to identify independent predictors of the uncontrolled seizure [26].

Finally, our study is not without limitations. First, the cross-sectional nature of the study may not provide adequate evidence of causality between poor seizure control and predictor variables. Second, due to self-report concerns, patients may understate socially undesirable activities like medication nonadherence. Lastly, our findings cannot be generalized to the whole patients with epilepsy found in Southwest Ethiopia because of two reasons. First, the study showed that about 54.6% of patients with epilepsy found in rural Ethiopia have poor treatment-seeking behavior because of social stigma, lack of knowledge, unfavorable attitude, and lack of social support [31]. Another study conducted in Ethiopia showed about 95.1% of patients with epilepsy seek treatment if there is a sudden loss of consciousness [15]. Second, our study was conducted in a single hospital found in Southwest Ethiopia.

## 5. Conclusions

Based on our study, more than half of the patients with epilepsy had uncontrolled seizures. Nonadherence to AEDs, number of seizure attacks before AEDs initiation, and head injury before seizure occurrence were predictors of uncontrolled seizure. AEDs adherence should be increased by an access to antiepileptic drugs without charge and attention should be given to patients with a history of head injury and the high number of seizure attacks/week before AEDs initiation. Moreover, we recommend researchers to do further longitudinal and interventional studies to provide adequate evidence about the cause-effect relationship between the predictor variables and seizure control.

## Figures and Tables

**Table 1:** Sociodemographic characteristics of epileptic patients on AEDs at MTUTH from March 10 to April 10, 2018.

Sociodemographic characteristic	Frequency (*N* = 143)

Age	
15–30	78 (54.6)
31–45	46 (32.2)
46 and above	19 13.3)

Sex	
Male	74 (51.7)
Female	69 (48.3)

Marital status	
Married	71 (49.7)
Single	63 (44.1)
Divorced	4 (2.8)
Widowed	5 (3.5)

Occupation	
Government employee	17 (11.9)
Farmers	41 (28.7)
Students	53 (37.1)
Merchants	24 (16.8)
Daily labors	8 (5.6)

Place of residence	
Rural	88 (61.5)
Urban	55 (38.5)

Level of education	
Not educated	15 (10.5)
Primary (1–8)	79 (55.2)
Secondary (9–12)	27 (18.9)
College/university	22 (15.4)

Monthly income	
Less than 500	35 (24.5)
500–1000	51 (35.7)
1000–2000	43 (30.1)
2000 and above	14 (9.7)

**Table 2:** Clinical information and seizure treatment outcome among epileptic patients on AEDs at MTUTH from March 10 to April 10, 2018.

Clinical information and determinants of prognostic factors	Frequency (%)
Status of treatment outcome	
Well-controlled	56 (39.2)
Poorly controlled	87 (60.8)

Types of epilepsy	
Generalized tonic-clonic seizure	111 (77.6)
Unclassified epilepsy	32 (22.4)

Time on AEDs	
2–5 years	120 (83.9)
5 years and above	23 (16.1)

Follow-up in the clinic	
2–5 years	115 (80.5)
5 years and above	28 (19.6)

Frequency of seizure attack/week before AEDs initiation	
<4	69 (48.2)
≥4	74 (52.8)

Comorbidity	
Yes	40 (28.0)
No	103 (72.0)

Types of comorbidity	
Psychiatric conditions	19 (13.3)
Hypertension	11 (7.7)
Diabetic mellitus	7 (4.9)
Heart failure	3 (2.1)

Triggering factors	
Yes	105 (73.4)
No	38 (26.6)

Types of triggering factors	
Emotional stress	63 (44.0)
Sleep deprivation	37 (25.9)
Missing medication	23 (16.1)
Heavy alcohol use	5 (3.9)
Chat and stimulant	4 (2.8)
Others	9 (6.3)

Time since seizure-free	
2–5 yrs	47 (32.9)
5 and above	9 (6.3)

Time to enter remission phase	
2–5 yrs	39 (27.3)
5 and above	17 (11.9)

History of head injury	
Yes	68 (47.5)
No	75 (52.4)

Time of head injury	
Before seizure occurrence	53 (37.0)
After seizure occurrence	15 (10.5)

EEG abnormality	
Yes	90
No	53

Others: dust, anger, high temperature, and headache. ECG: electrocardiogram.

**Table 3:** Antiepileptic drugs and associated adverse effects among epileptic patients on AEDs at MTUTH from March 10 to April 10, 2018.

Antiepileptic drug	Adverse effects	Frequency (%)
*Types of AEDs used*	Phenobarbitone alone	90 (62.9)
	Phenytoin alone	23 (16.1)
	Phenobarbitone plus phenytoin	17 (11.9)
	Phenobarbitone plus carbamazepine	9 (6.3)
	Carbamazepine plus phenytoin	4 (2.8)
*AEDs prescribed as initial therapy*	Phenobarbitone	117 (81.8)
	Phenytoin	22 (15.4)
	Carbamazepine	4 (2.8)
*AEDs prescribed as add-on therapy*	Phenytoin	21 (14.7)
	Carbamazepine	12 (8.4)
*Polytherapy with AEDs*	Yes	30 (21.0)
	No	113 (79.0)
*Polytherapy irrespective of AEDs*	Yes	70 (48.9)
	No	73 (51.1)
*Antiepileptic-related adverse effects*	Yes	104 (72.7)
	No	39 (27.3)
*Types of antiepileptic associated adverse effects*	Sedation	78 (54.5%)
	Confusion	10 (7.0)
	Weakness	9 (6.3)
	Gingival hyperplasia	9 (6.3)
	Rash	7 (4.9)
	Blurred vision	6 (4.2)
	GI irritation	4 (2.8)

**Table 4:** Reasons for nonadherence among epileptic patients on AEDs at MTUTH from March 10 to April 10, 2018.

Reasons for no adherence	Reasons for nonadherence	Frequency	Percent
*Patient-related factors*	You cannot afford to buy the medications	44	40.7
	The schedule of your work makes it impossible	23	21.3
	Forget to take medications	11	10.2
	Medication-related side effect	16	14.8
*Medication-related factor*	Misunderstanding of instructions about how to take the drugs	11	10.2
*Health system-related factor*	Lack of free medicine supply	6	5.6
	Lack of education about AEDs	34	31.5
	Poor relationship between patient and physician	8	7.4
*Condition-related factors*	Forgetfulness	47	43.5
	Memory deficits	7	6.5
	High frequency of seizure	3	2.8
	Duration and previous treatment failure	1	0.93
*Socioeconomic-related factors*	Long distance from treatment setting	30	27.8
	High cost of medication	21	19.4

**Table 5:** Predictors of poorly controlled seizure among epileptic patients on AEDs at MTUTH from March 10 to April 10, 2018.

Variables affecting treatment outcome	Seizure treatment outcome		AOR (95% CI)	*p* value
	Controlled frequency (%)	Noncontrolled frequency (%)		
Level of adherence				
High adherence	25 (71.4%)	10 (28.6%)	1.00	1.00
Medium adherence	12 (50%)	12 (50%)	5.4 (1.52–19.23)	0.009
Low adherence	19 (22.6%)	65 (77.4%)	8.16 (3.04–21.90)	0.00 1

History of head injury				
Yes (68)	19 (28%)	49 (72%)	2.4 (1.088–5.314)	0.03
No (75)	37 (49.3%)	38 (50.7)	1.00	1.00

History of head injury				
Before seizure occurrence	10 (18.9%)	43 (81.1%)	4.90 (1.25–19.27)	0.02
After seizure occurrence	9 (60%)	6 (40%)	1.00	1.00

Frequency of seizure attacks per week before AEDs initiation				
<3 times	43 (62.3%)	26 (37.7%)	1.00	1.00
>4 times	15 (20%)	59 (79.7%)	1.98 (1.05 5.98)	0.012

Marital status				
Married	26 (36.6%)	45 (63.4%)	1.00	1.00
Single	26 (41.3%)	37 (58.7%)	0.64 (0.27–1.51)	0.31
Divorced	0	4	9.9 (0.05–4.45)	0.99
Widowed	4 (80%)	1 (20%)	0.08 (0.01–0.93)	0.04

Monthly income				
>2000	8 (57.1%)	6 (42.9%	1.00	1.00
1000–2000	15 (34.9%)	28 (65.1%)	2.09 (0.71–7.51)	0.246
500–1000	15 (48.4%)	16 (51.6%)	1.62 (0.29–4.57)	0.384
Less than 500	9 (36%)	16 (64%)	2.03 (0.32–1.99)	0.236

Educational status				
College/university	7 (31.8%)	15 (68.2%)	1.00	1.00
Secondary (9–12)	10 (37%)	17 (63)	1.05 (0.12–1.86)	0.583
Primary (1–8)	31 (39.2%)	48 (60.8%)	2.20 (0.65–2.87)	0.526
Not educated	8 (53.3%)	7 (46.7%)	1.02 (0.11–1.58)	0.095

## Data Availability

The raw data supporting the results reported in this article are available upon reasonable request by contacting the corresponding author AZ.

## Abbreviations

AOR: Adjusted odds ratio
AEDs: Antiepileptic drugs
GTCS: Generalized tonic-clonic seizure
GNP: Gross national product
MTUTH: Mizan-Tepi University Teaching Hospital
SPSS: Statistical Package for Social Science
WHA: World Health Assembly
WHO: World Health Organization.
